# Dense breast stromal tissue shows greatly increased concentration of breast epithelium but no increase in its proliferative activity

**DOI:** 10.1186/bcr1408

**Published:** 2006-04-28

**Authors:** Debra Hawes, Susan Downey, Celeste Leigh Pearce, Sue Bartow, Peggy Wan, Malcolm C Pike, Anna H Wu

**Affiliations:** 1 Department of Pathology, Keck School of Medicine, University of Southern California, 2011 Zonal Avenue, Los Angeles, CA 90089, USA; 2 Department of Surgery, Keck School of Medicine, University of Southern California, 1510 San Pablo Street, Los Angeles, CA 90033, USA; 3 Department of Preventive Medicine, Keck School of Medicine, University of Southern California/Norris Comprehensive Cancer Center, 1441 Eastlake Avenue, Los Angeles, CA 90033, USA; 4 107 Stark Mesa, Carbondale, CO 81623, USA

## Abstract

**Introduction:**

Increased mammographic density is a strong risk factor for breast cancer. The reasons for this are not clear; two obvious possibilities are increased epithelial cell proliferation in mammographically dense areas and increased breast epithelium in women with mammographically dense breasts. We addressed this question by studying the number of epithelial cells in terminal duct lobular units (TDLUs) and in ducts, and their proliferation rates, as they related to local breast densities defined histologically within individual women.

**Method:**

We studied deep breast tissue away from subcutaneous fat obtained from 12 healthy women undergoing reduction mammoplasty. A slide from each specimen was stained with the cell-proliferation marker MIB1. Each slide was divided into (sets of) areas of low, medium and high density of connective tissue (CT; highly correlated with mammographic densities). Within each of the areas, the numbers of epithelial cells in TDLUs and ducts, and the numbers MIB1 positive, were counted.

**Results:**

The relative concentration (RC) of epithelial cells in high compared with low CT density areas was 12.3 (95% confidence interval (CI) 10.9 to 13.8) in TDLUs and 34.1 (95% CI 26.9 to 43.2) in ducts. There was a much smaller difference between medium and low CT density areas: RC = 1.4 (95% CI 1.2 to 1.6) in TDLUs and 1.9 (95% CI 1.5 to 2.3) in ducts. The relative mitotic rate (RMR; MIB1 positive) of epithelial cells in high compared with low CT density areas was 0.59 (95% CI 0.53 to 0.66) in TDLUs and 0.65 (95% CI 0.53 to 0.79) in ducts; the figures for the comparison of medium with low CT density areas were 0.58 (95% CI 0.48 to 0.70) in TDLUs and 0.66 (95% CI 0.44 to 0.97) in ducts.

**Conclusion:**

Breast epithelial cells are overwhelmingly concentrated in high CT density areas. Their proliferation rate in areas of high and medium CT density is lower than that in low CT density areas. The increased breast cancer risk associated with increased mammographic densities may simply be a reflection of increased epithelial cell numbers. Why epithelium is concentrated in high CT density areas remains to be explained.

## Introduction

On a mammogram, fat appears radiolucent or dark, whereas stromal and epithelial tissue appears radio-dense or white. The amount of mammographic density is a strong independent predictor of breast cancer risk [1,2]. The biological basis for this increased risk is poorly understood. A critical question is whether densities are directly related to risk or are simply a marker of risk. We addressed this question recently by studying the location of small ductal carcinoma *in situ *(DCIS) lesions as revealed by microcalcifications, and showed that such DCIS occurs overwhelmingly in the mammographically dense areas of the breast [3]. Most DCIS lesions in our study occurred in the lateral-superior quadrant, as has been found in previous studies [4], and 'correlated strongly with the average percentage density in the different mammographic quadrants' [3]. Pre-DCIS mammograms that were taken on average about two years previously showed that the areas subsequently exhibiting DCIS were clearly dense at the time of the earlier mammogram, and this suggests that this relationship was not brought about by the presence of the DCIS. The reasons for these findings are not clear; two obvious possibilities are increased epithelial cell proliferation in mammographically dense areas of the breast and increased breast epithelium in women with mammographically dense breasts. Two groups have investigated the relationship between the amount of mammographic density of a woman and the amount of her breast epithelial tissue [5,6]. Alowami and colleagues [5] used tissue obtained from biopsies investigating breast lesions that were subsequently diagnosed as benign or pre-invasive breast disease; they studied tissue 'distant from the diagnostic lesion' without reference to its location as regards mammographic density (that is, 'random' tissue). They found that the median density of duct lobular units was 28% higher in breasts whose overall mammographic density was 50% or more (*n *= 27) than in breasts whose overall mammographic density was less than 25% (*n *= 35); this result was not statistically significant and the result was described as showing 'no difference in the density of epithelial components' [5]. Li and colleagues [6] also found in their much larger study (*n *= 236) of 'random' breast tissue collected from normal women by Bartow and colleagues [7] in their autopsy study of accidental deaths in New Mexico that women with high mammographic density had greater amounts of epithelial tissue (as measured by area of epithelial nuclear staining) and the result was highly statistically significant. Breast epithelial proliferation rates as they relate to mammographic densities in healthy women have not been well studied [8]. We have addressed these questions by studying the number of epithelial cells in terminal duct lobular units (TDLUs) and in breast ducts, and their respective proliferation rates as they relate to local histological breast densities within individual women.

## Materials and methods

We retrospectively identified 15 consecutive healthy women who had undergone a reduction mammoplasty performed by one of us (SD) at the University of Southern California medical facilities. The study protocol was approved by the Institutional Review Board of the University of Southern California School of Medicine.

For each participant we obtained the formalin-fixed paraffin-embedded block of tissue that had been routinely processed and saved from her surgery. A single slide was cut from each block and stained with the proliferation marker MIB1 (BioGenex Laboratories, San Ramon, CA, USA). The slides were prepared in accordance with our previously published protocol [9]; the chromogen used was 3,3'-diaminobenzidine tetrahydrochloride (DAB). On microscopic examination one of the slides contained skin and two other slides showed areas of disintegration; all three were deemed unsuitable for study.

**Figure 1:**
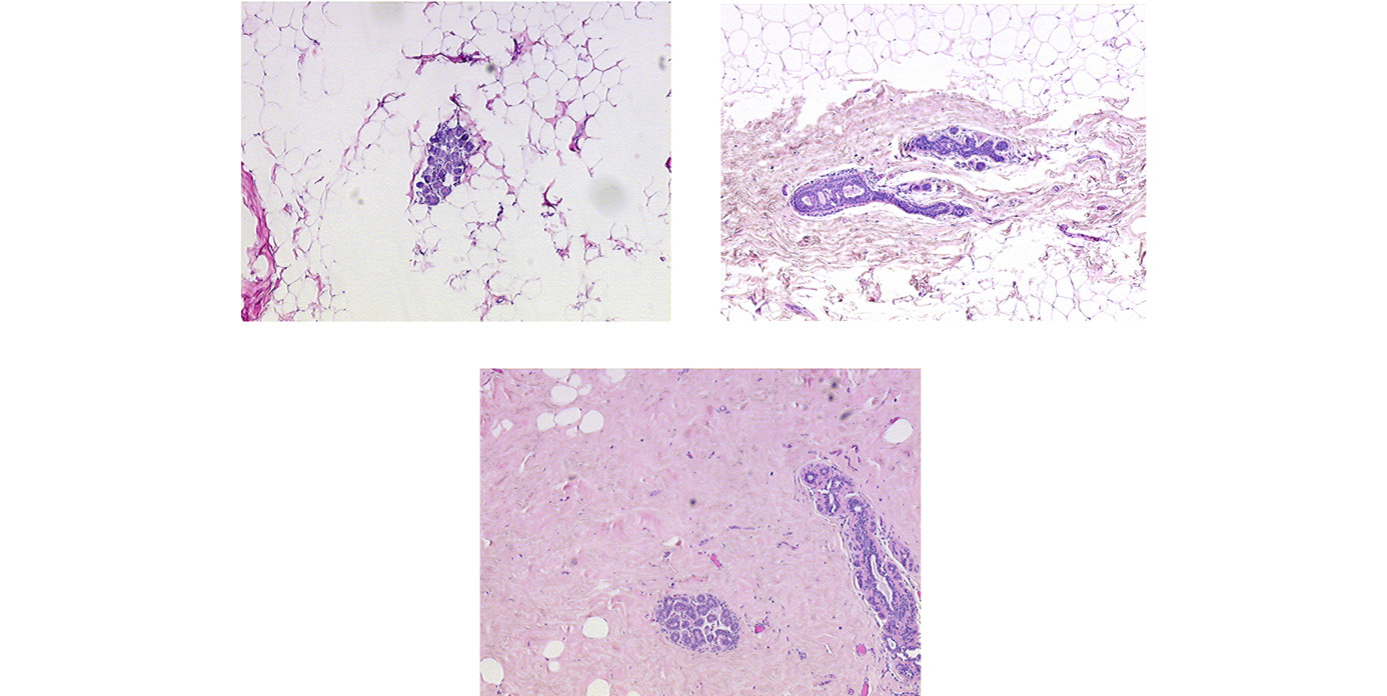
Example of areas of low, medium (upper right) and high (lower center) CT density.

Each of the remaining 12 slides was divided into (sets of) areas of low, medium and high density of connective tissue (CT) (highly correlated with densities as defined by mammographic criteria [10]); see Figure 1. The total size of each of the three areas (in μm^2^), and within each of the three areas the numbers of epithelial cells in TDLUs and ducts and the numbers that were MIB1 positive, were counted with the help of an automated microscope system that digitized the images and permitted the outlining of relevant areas on a high-resolution computer screen (ACIS II; Clarient, Inc., San Juan Capistrano, CA, USA). The total numbers of epithelial cells in different outlined areas within the CT density-defined areas was then automatically counted by the ACIS II nuclear counting software program, which is based on color identification. Hematoxylin was used to counterstain the MIB1-negative nuclei blue, and the DAB chromogen marked the MIB1-positive nuclei brown. The software calculated the numbers of MIB1-negative and MIB1-positive cells on the basis of these color differences.

### Statistical analysis

For each slide, and separately for TDLU and ductal cells, three sets of values were obtained: first, the areas of the slide classified as being of low, medium or high CT density (*a*_L_, *a*_M _and *a*_H _in μm^2^); second, the numbers of epithelial cells within these areas (*t*_L_, *t*_M _and *t*_H_); and third, the numbers of these epithelial cells staining positive for MIB1 (*n*_L_, *n*_M _and *n*_H_). On the null hypothesis of no association between the *t*'s and the *a*'s – that is, no association between the numbers of epithelial cells and the CT density of the local tissue – the expected value of the *t*'s is simply proportional to the related *a*'s, so that, for example, the expected value of *t*_H _is (*t*_L _+ *t*_M _+ *t*_H_) × *a*_H_/(*a*_L _+ *a*_M _+ *a*_H_). Similarly, on the null hypothesis of no association between MIB1 positivity as a proportion of epithelial cells and the CT density of the local tissue, the expected value of the *n*'s is simply proportional to the related *t*'s, so that, for example, the expected value of *n*_H _is (*n*_L _+ *n*_M _+ *n*_H_) × *t*_H_/(*t*_L _+ *t*_M _+ *t*_H_). We analyzed these data with standard statistical software as implemented in the STATA statistical software package (procedure cs; Stata Corporation, Austin, TX, USA); the ratios of epithelial concentration (cells per unit area) and the ratios of proportions of epithelial cells staining positive for MIB1 are the measures of effect. All statistical significance levels (*p *values) quoted are two-sided.

## Results

The 12 subjects included in the analysis were aged 18 to 60 years with a median age of 33 years; only one subject was aged 50 years or older.

Areas of the slides of low CT density comprised on average 44% of the total of areas of low plus medium plus high CT density (*a*_L_/(*a*_L _+ *a*_M _+ *a*_H_)), whereas areas of high CT density comprised on average 35% of the total area (*a*_H_/(*a*_L _+ *a*_M _+ *a*_H_)).

**Figure 2:**
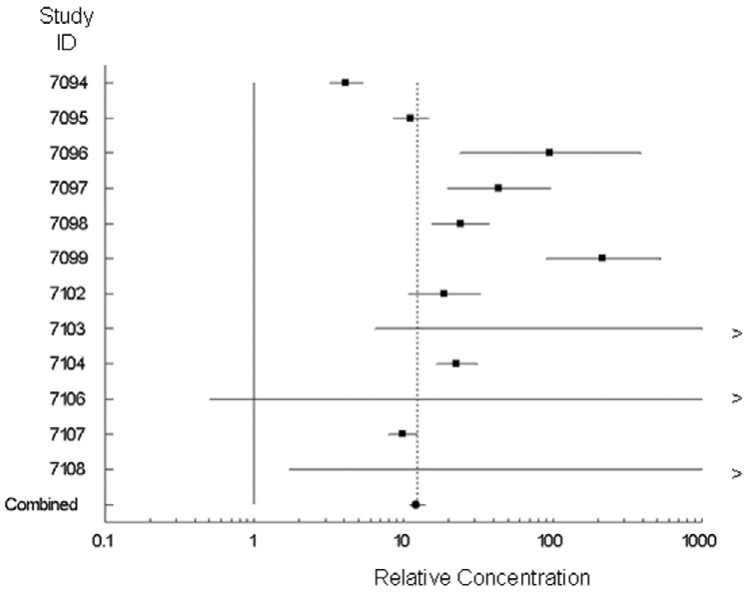
RCs (with 95% CIs) of TDLU epithelial cells in high and low CT areas.

Table 1 shows the summary relative concentrations (RCs; ratios of cells per unit area) of epithelial cells in the three areas defined by CT density separately for TDLU cells and for ductal cells. The concentration of TDLU epithelial cells is slightly greater in the areas of medium CT density than in the areas of low CT density (RC = 1.4, 95% confidence interval (CI) 1.2 to 1.6; *p *< 0.001) but is much greater in the areas of high CT density (RC = 12.3, 95% CI 10.8 to 13.8; *p *< 0.001). The TDLU results for the individual slides (women) comparing areas of high CT density with areas of low CT density are shown in Figure 2. Although the results from individual subjects do differ somewhat, the RCs were not correlated with age (the only variable available on these women) and the summary RC seems to be a fair representation of the overall results. The results for ducts were similar.

**Figure 3:**
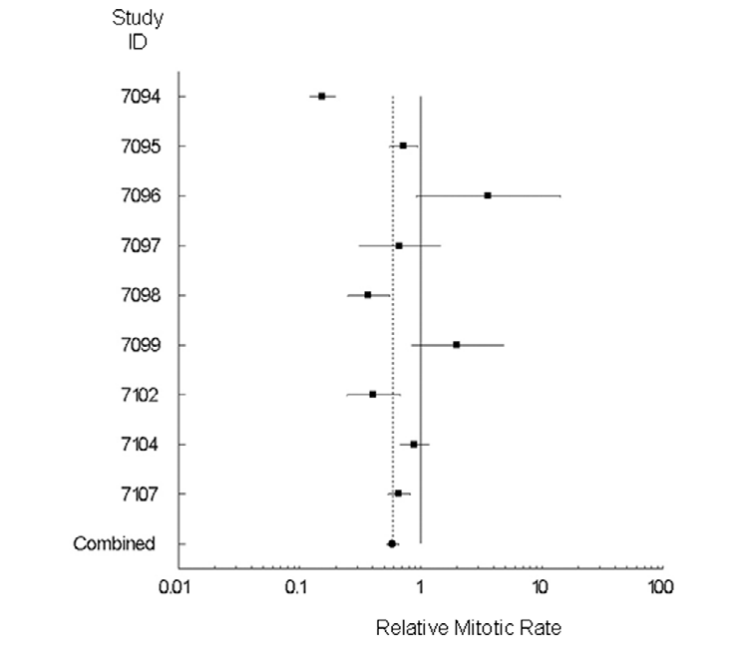
RMRs (with 95% CIs) of TDLU epithelial cells in high and low CT areas.

Table 2 shows the summary relative mitotic rates (RMRs) of epithelial cells staining MIB1 positive in the three areas defined by CT density separately for TDLU cells and for ductal cells. The proportion of TDLU epithelial cells staining MIB1 positive is statistically significantly less (RMR ≈ 0.6) both in the areas of medium CT density (*p *< 0.001) and in the areas of high CT density (*p *< 0.001) than in the areas of low CT density. The median MIB1-positive proportion was about 4%. Almost all the women in this study were premenopausal on the basis of their age; this figure is close to the Ki67 figure of 4.5% given for healthy premenopausal women in the study of Hargreaves and colleagues [11]. The TDLU results for the individual slides (women) comparing areas of high CT density with areas of low CT density are shown in Figure 3. Again, although the results from individual subjects do differ somewhat, the RMRs were not correlated with age (the only variable available on these women) and the summary RMR seems to be a fair representation of the overall results. The results for ducts were again similar. There was no difference in the proliferation rates of epithelial cells in TDLUs and ducts within the same CT density area of individual women (RMR = 1.01, 95% CI 0.98 to 1.04; *p *= 0.42).

More details of the results are provided in the Additional file.

## Discussion

Mammographic density is a very strong risk factor for breast cancer. The two groups of investigators [5,6] that studied random biopsies (single slides) from women with different mammographic densities found that the extent of mammographic densities was most strongly correlated with the amount of collagen on the slide. A weaker correlation was found with the amount of epithelial tissue. The findings reported here suggest that the relation between the extent of mammographic density and the amount of epithelial tissue is directly related to the increased concentration of collagen (the main component of 'connective tissue' as shown by collagen staining; see Figure 1) in women with high mammographic densities, because breast epithelium is overwhelmingly confined to areas of high CT density. In the earlier studies of random biopsies [5,6] the weaker relationship between mammographic density and epithelium concentration than between mammographic density and collagen concentration could be simply due to the much greater statistical variability of epithelial tissue in a random slide than one would see for collagen, which occupies a much greater extent of the slide. These results suggest that the increasing breast cancer risk associated with increasing mammographic density might be simply a reflection of more breast epithelial tissue.

We found that the proliferation rate of epithelial cells in areas of high CT density was much lower than in areas of low CT density, arguing against the possibility that dense stroma has a growth factor role in the increased breast cancer risk of women with mammographically dense breasts. In the study of Stomper and colleagues [8], comparison was made between single biopsies of either fat or dense areas in different women; they found no difference in the proliferation rates in the dense and fat areas. Further work is warranted but there is clearly no evidence that areas of high CT density are associated with increased proliferation.

Our results were obtained by conducting a comprehensive count of all the cells in each slide per subject (instead of counting a selected region) and allowed the comparison of proliferation rates in areas of differing CT density within an individual. This permitted us to control completely automatically for factors such as age, menopausal status, or time in the menstrual cycle in the analysis. This gave us great statistical power so that highly statistically significant results could be obtained even with small numbers of subjects.

This study used tissue obtained at reduction mammoplasty performed on women with large breasts. We do not believe that this affects the validity of our findings because the tissue samples were taken deep in the breast away from the subcutaneous fat, but this requires confirmation in future studies. Further studies are also needed relating the CT densities to such risk factors as parity and to understand the biology of the relationship between CT densities and breast epithelium.

## Conclusion

The basis of the strong relationship between mammographic density and breast cancer risk may be simply that mammographically dense breasts contain more breast epithelial tissue. Why breast epithelial tissue should be associated with CT densities is not known. Does breast epithelium induce densities? Alternatively, can breast epithelium effectively survive only in areas of densities? Understanding the nature of the interaction between dense CT stroma and epithelial tissue should be a major focus of breast cancer research.

## Abbreviations

*a*_H_, *a*_L_, *a*_M _= the areas of the slide classified as being of high, low and medium CT density (in μm^2^); CI = confidence interval; CT = connective tissue; DAB = 3,3'-diaminobenzidine tetrahydrochloride; DCIS = ductal carcinoma *in situ*; *n*_H_, *n*_L_, *n*_M _= the numbers of epithelial cells staining positive for MIB1 within high, low and medium CT density areas; RC = relative concentration; RMR = relative mitotic rate; TDLU = terminal duct lobular unit; *t*_H_, *t*_L_, *t*_M _= the numbers of epithelial cells within high, low and medium CT density areas;

## Competing interests

The authors declare that they have no competing interests.

## Authors' contributions

DH, AHW, CLP and MCP participated in the design of the study. DH supervised the preparation of the slides and analyzed the slides with the ACIS II system. SD performed all the reduction mammoplasties that provided the tissues used in this analysis and consulted on the tissue obtained from reduction mammoplasties. SB consulted on the interpretation of the results and provided insight into the relationship between mammographic densities and tissue characteristics. CLP coordinated the study. MCP supervised the statistical analysis which was carried out by PW. AHW, CLP and MCP conceived of the study. MCP, DH and AHW drafted the manuscript; all authors read and approved the final manuscript.

## Supplementary Material

Additional File 1 A Word file containing two tables of detailed results from this study. Click here for file

## Figures and Tables

**Table 1:** Relation between relative concentration of epithelial cells and connective tissue density

CT density	RC	95% CI	*p*
TDLUs			
Low	1.0		
Medium	1.4	1.2–1.6	<0.001
High	12.3	10.9–13.8	<0.001
Ducts			
Low	1.0		
Medium	1.9	1.5–2.3	<0.001
High	34.1	26.9–43.2	<0.001

CI, confidence interval; CT, connective tissue; RC, relative concentration (per unit area); TDLUs, terminal duct lobular units.

**Table 2:** Relation between relative mitotic rate (MIB1 positive) of epithelial cells and connective tissue density

CT density	RMR	95% CI	*p*
TDLUs			
Low	1.00		
Medium	0.58	0.48–0.70	<0.001
High	0.59	0.53–0.66	<0.001
Ducts			
Low	1.00		
Medium	0.66	0.44–0.97	0.035
High	0.65	0.53–0.79	<0.001

CI, confidence interval; CT, connective tissue; RMR, relative mitotic rate; TDLUs, terminal duct lobular units.
