# Supplementary material for: Dense breast stromal tissue shows greatly increased concentration of breast epithelium but no increase in its proliferative activity
Source: Breast Cancer Res. 2006 Apr 28;8(2):R24. doi: 10.1186/bcr1408 (PMC1557710; doi:10.1186/bcr1408)
Supplement: Additional File 1 — A Word file containing two tables of detailed results from this study. [file bcr1408-S1.doc]

Appendix

|  | | | | | | |  |
| --- | --- | --- | --- | --- | --- | --- | --- |
| Table A. TDLU cells results | | | | | | |  |
|  | | | | | | |  |
|  |  |  | | | | |  |
|  |  |  | Ct Density | | | |  |
| ID | Age |  | Low | | Medium | High | |
|  |  |  | |  |  |  | |
| 7094 | 33 | Area (a; µm^2^×10^6^) | | 140.2 | 76.5 | 136.7 | |
|  |  | # cells (t) | | 346 | 180 | 8955 | |
|  |  | # MIB1 +ve (n) | | 71 | 17 | 285 | |
| 7095 | 40 | Area | | 89.5 | 103.6 | 40.8 | |
|  |  | # cells | | 1119 | 828 | 7918 | |
|  |  | # MIB1 +ve | | 61 | 29 | 312 | |
| 7096 | 30 | Area | | 336.6 | 7.2 | 283.5 | |
|  |  | # cells | | 57 | 488 | 1278 | |
|  |  | # MIB1 +ve | | 2 | 25 | 162 | |
| 7097 | 48 | Area | | 300.0 | 206.5 | 47.2 | |
|  |  | # cells | | 133 | 1765 | 1356 | |
|  |  | # MIB1 + ve | | 7 | 65 | 48 | |
| 7098 | 60 | Area | | 340.3 | 190.4 | 38.3 | |
|  |  | # cells | | 119 | 171 | 865 | |
|  |  | # MIB1 +ve | | 27 | 37 | 73 | |
| 7099 | 35 | Area | | 179.5 | 89.0 | 4.2 | |
|  |  | # cells | | 268 | 0 | 644 | |
|  |  | # MIB1 +ve | | 6 | 0 | 29 | |
| 7102 | 24 | Area | | 284.8 | 184.9 | 119.6 | |
|  |  | # cells | | 77 | 168 | 1496 | |
|  |  | # MIB1 +ve | | 14 | 10 | 111 | |
| 7103 | 38 | Area | | 76.3 | 47.9 | 142.2 | |
|  |  | # cells | | 0 | 0 | 1304 | |
|  |  | # MIB1 +ve | | 0 | 0 | 46 | |
| 7104 | 24 | Area | | 193.8 | 0 | 193.3 | |
|  |  | # cells | | 184 | 0 | 4665 | |
|  |  | # MIB1 +ve | | 41 | 0 | 927 | |
| 7106 | 21 | Area | | 44.1 | 30.7 | 407.6 | |
|  |  | # cells | | 0 | 0 | 583 | |
|  |  | # MIB1 +ve | | 0 | 0 | 18 | |
| 7107 | 28 | Area | | 93.0 | 68.1 | 93.6 | |
|  |  | # cells | | 1293 | 2002 | 19513 | |
|  |  | # MIB1 +ve | | 91 | 70 | 907 | |
| 7108 | 18 | Area | | 148.3 | 0.0 | 205.5 | |
|  |  | # cells | | 0 | 0 | 235 | |
|  |  | # MIB1 +ve | | 0 | 0 | 9 | |
|  |  |  | |  |  |  | |

|  | | | | | | | |  | | |
| --- | --- | --- | --- | --- | --- | --- | --- | --- | --- | --- |
| Table B. Duct cells results | | | | | | | | |  | |
|  | | | | | | | | |  | |
|  |  |  | | | | | |  | | |
|  |  | | ct Density | | | | | |  | |
| ID | Age |  | | Low | | Medium | High | |  | |
|  |  |  | | |  |  |  | | | |
| 7094 | 33 | Area | | | 140.2 | 76.5 | 136.7 | | |  |
|  |  | # cells | | | 113 | 0 | 1786 | | |  |
|  |  | # MIB1 +ve | | | 18 | 0 | 207 | | |  |
| 7095 | 40 | Area | | | 89.5 | 103.6 | 40.8 | | |  |
|  |  | # cells | | | 14 | 752 | 793 | | |  |
|  |  | # MIB1 +ve | | | 0 | 28 | 21 | | |  |
| 7096 | 30 | Area | | | 336.6 | 7.2 | 283.5 | | |  |
|  |  | # cells | | | 36 | 292 | 3995 | | |  |
|  |  | # MIB1 +ve | | | 1 | 39 | 554 | | |  |
| 7097 | 48 | Area | | | 300.0 | 206.5 | 47.2 | | |  |
|  |  | # cells | | | 594 | 914 | 2419 | | |  |
|  |  | # MIB1 +ve | | | 45 | 27 | 42 | | |  |
| 7098 | 60 | Area | | | 340.3 | 190.4 | 38.3 | | |  |
|  |  | # cells | | | 903 | 25 | 1981 | | |  |
|  |  | # MIB1 +ve | | | 51 | 3 | 98 | | |  |
| 7099 | 35 | Area | | | 179.5 | 89.0 | 4.2 | | |  |
|  |  | # cells | | | 31 | 447 | 300 | | |  |
|  |  | # MIB1 +ve | | | 0 | 12 | 6 | | |  |
| 7102 | 24 | Area | | | 284.8 | 184.9 | 119.6 | | |  |
|  |  | # cells | | | 5 | 504 | 2322 | | |  |
|  |  | # MIB1 +ve | | | 0 | 26 | 202 | | |  |
| 7103 | 38 | Area | | | 76.3 | 47.9 | 142.2 | | |  |
|  |  | # cells | | | 0 | 0 | 888 | | |  |
|  |  | # MIB1 +ve | | | 0 | 0 | 40 | | |  |
| 7104 | 24 | Area | | | 193.8 | 0.0 | 193.3 | | |  |
|  |  | # cells | | | 0 | 0 | 3361 | | |  |
|  |  | # MIB1 +ve | | | 0 | 0 | 523 | | |  |
| 7106 | 21 | Area | | | 44.1 | 30.7 | 407.6 | | |  |
|  |  | # cells | | | 0 | 0 | 3051 | | |  |
|  |  | # MIB1 +ve | | | 0 | 0 | 70 | | |  |
| 7107 | 28 | Area | | | 93.0 | 68.1 | 93.6 | | |  |
|  |  | # cells | | | 84 | 0 | 130 | | |  |
|  |  | # MIB1 +ve | | | 17 | 0 | 17 | | |  |
| 7108 | 18 | Area | | | 148.3 | 0 | 205.5 | | |  |
|  |  | # cells | | | 0 | 0 | 670 | | |  |
|  |  | # MIB1 +ve | | | 0 | 0 | 27 | | |  |
|  |  |  | | |  |  |  | | |  |
